# Supplementary material for: Prophage and metabolic determinants of Staphylococcus aureus survival to vancomycin identified via TraDIS screening
Source: mSystems. 2026 Sep 16;11(10):e00734-26. doi: 10.1128/msystems.00734-26 (PMC13712775; doi:10.1128/msystems.00734-26)
Supplement: Supplementary file 1 [file msystems.00734-26-s0005.docx]

**Supplementary Figure Legends**

**Figure S1. Impact of selected USA300 transposon mutants on growth during exposure to vancomycin at the MIC.** Growth of defined mutants from the *S. aureus* USA300 JE2 Nebraska Transposon Mutant Library in **(A)** the absence of vancomycin and (B) the presence of vancomycin at its MIC, i.e. 2 µg/mL. Growth was monitored by OD₆₀₀. Data represent mean and standard error from three biological replicates.

**Figure S2.** **Impact of selected USA300 transposon mutants on the binding of vancomycin**. **(A)** Representative histogram showing the distribution of FITC fluorescence intensity (FITC-A) for WT and transposon mutant strains following 10 min exposure to 2 µg/mL vancomycin-NBD (i.e. the MIC of vancomycin-NBD against the JE2 WT strain). Each curve represents an individual strain, illustrating vancomycin binding compared to the unlabeled WT control. **(B)** Percentage of FITC-positive cells measured by flow cytometry after 10 min incubation in 2 µg/mL FITC-labelled vancomycin. Three biological replicas shown.

**Figure S3. Impact of medium and phase of growth on vancomycin efficacy against selected USA300 transposon mutants.** Growth of defined mutants from the *S. aureus* USA300 JE2 Nebraska Transposon Mutant Library was monitored by OD₆₀₀ under (A) exponential-phase conditions in Mueller-Hinton (MH) broth and (B) stationary-phase conditions in LB broth. Data represent the mean and standard error from three biological replicates.

**Supplementary File Legends**

**File S1. Transposon insertion counts and differential analysis for cultures exposed to vancomycin at the MIC.** Genome-wide transposon insertion counts per gene, log2 fold change, and associated p values for S0385 transposon library cultures incubated for 5 h in the presence of vancomycin at the wild-type MIC.

**File S2. Transposon insertion counts for LB-grown control cultures.** Genome-wide transposon insertion counts per gene for S0385 transposon library cultures grown in LB only, derived from the same cultures prior to vancomycin exposure.

**File S3. List of genes associated with loss of survival at the vancomycin MIC.** Genes identified by TraDIS as significantly enriched in MIC-non-surviving cultures are grouped by predicted biological function based on genome annotation. Gene names are shown where available; otherwise, locus tags are provided. Genes were considered significant based on the following criteria: log₂FC > 1.5, a statistically significant p value, ≤3 transposon insertions in genes required for survival at the vancomycin MIC (File S1), and ≥4 insertions in at least one replicate under LB growth conditions, indicating that the gene is not required for growth in LB (File S2).

**File S4. Predicted Pfam domains identified in the protein sequence using KEGG annotation.** Pfam domains detected in proteins from predicted prophage regions. The table shows the prophage region, locus tag, predicted protein annotation, identified Pfam motifs, corresponding E-values, and functional notes. Pfam domain annotations were retrieved from KEGG database resources. An E-value cutoff of 0.01 was applied, and only hits corresponding to bacterial or bacteriophage proteins were considered.

**File S5. Strains from Nebraska Transposon Mutant Library (NTML) - collection generated in the *S. aureus* USA300 background used in this study.**

**File S6. Minimal inhibitory concentration (MIC) for USA300 NTML strains.**

**File S7. List of primers.** All primers were designed for *S. aureus* S0385.
